# Exploration of symptom clusters during hemodialysis and symptom network analysis of older maintenance hemodialysis patients: a cross-sectional study

**DOI:** 10.1186/s12882-023-03176-4

**Published:** 2023-04-27

**Authors:** Mingyao Zhou, Xiaoxin Gu, Kangyao Cheng, Yin Wang, Nina Zhang

**Affiliations:** 1 School of Nursing, Shanghai University of Traditional Chinese Medicine, No.1200 Cailun Road, Pudong New District, Shanghai, 201203 China; 2 Hemodialysis Room, Shanghai Sixth People’s Hospital, Shanghai Jiaotong University, No.600 Yishan Road, Xuhui District, Shanghai, 201306 China

**Keywords:** Maintenance hemodialysis in older patients, Symptom burden, Symptom cluster, Symptom network, Core symptoms, Influencing factors

## Abstract

**Background:**

Symptom networks can provide empirical evidence for the development of personalized and precise symptom management strategies. However, few studies have established networks of symptoms experienced by older patients on maintenance hemodialysis. Our goal was to examine the type of symptom clusters of older maintenance hemodialysis patients during dialysis and construct a symptom network to understand the symptom characteristics of this population.

**Methods:**

The modified Dialysis Symptom Index was used for a cross-sectional survey. Network analysis was used to analyze the symptom network and node characteristics, and factor analysis was used to examine symptom clusters.

**Results:**

A total of 167 participants were included in this study. The participants included 111 men and 56 women with a mean age of 70.05 ± 7.40. The symptom burdens with the highest scores were dry skin, dry mouth, itching, and trouble staying asleep. Five symptom clusters were obtained from exploratory factor analysis, of which the clusters with the most severe symptom burdens were the gastrointestinal discomfort symptom cluster, sleep disorder symptom cluster, skin discomfort symptom cluster, and mood symptom cluster. Based on centrality markers, it could be seen that feeling nervous and trouble staying asleep had the highest strength, and feeling nervous and feeling irritable had the highest closeness and betweenness.

**Conclusions:**

Hemodialysis patients have a severe symptom burden and multiple symptom clusters. Dry skin, itching, and dry mouth are sentinel symptoms in the network model; feeling nervous and trouble staying asleep are core symptoms of patients; feeling nervous and feeling irritable are bridge symptoms in this symptom network model. Clinical staff can formulate precise and efficient symptom management protocols for patients by using the synergistic effects of symptoms in the symptom clusters based on sentinel symptoms, core symptoms, and bridge symptoms.

**Supplementary Information:**

The online version contains supplementary material available at 10.1186/s12882-023-03176-4.

## Background

End-stage renal disease (ESRD) is a disease that leads to a rapidly increasing global health and medical burdens, with studies showing mortality rates as high as 30% within the first year of transition from chronic kidney disease (CKD) to ESRD [1]. ESRD patients mainly rely on renal replacement therapy with renal transplantation or dialysis. From 2003 to 2016, the prevalence of ESRD was relatively stable in various countries [2], and according to the ISN 2019 GKHA survey [3], the average number of people treated globally for ESRD was 759 per million people per year, with the largest proportion in the USA (29%), followed by Japan (13%) and Brazil (7%). Most countries consider dialysis the first-choice therapy. Worldwide, hemodialysis (HD) is the most common form of dialysis [1]. In most countries, more than 80% of chronic dialysis patients are treated with HD [2, 4]. Although HD therapy is widely used, the internal environment of the body changes greatly after the treatment. Karasneh et al. [5] found that patients with maintenance hemodialysis (MHD) had on average 13 discomfort symptoms after dialysis [5]. The results of several relevant studies have shown that the prevalence of symptoms ranges from 40.7% to 92.3% in HD patients [6–9]. Among the physical symptoms, skin itching, fatigue, sleep disorder, and other symptoms are the most prominent [10–13], while among the psychological symptoms, anxiety and depression are particularly common [10, 14, 15]. Especially in older patients, with increasing age, the symptoms occur more often and are more severe, thereby negatively affecting the patients’ quality of life. For example, fatigue may lead to the occurrence of cardiovascular disease and even death of patients [16], while skin itching can lead to the decline of sleep quality of patients and bring negative emotions to patients [17, 18], so there is an urgent need for comprehensive and efficient symptom management programs to alleviate the occurrence of symptoms.

Current research on symptom management of patients with MHD has mostly focused on individual symptoms, but patients typically experience multiple concurrent interrelated symptoms (i.e., symptom clusters). These interrelated symptoms may often share one or more potential mechanisms. By identifying the synergy between symptoms, we can carry out the classification and centralized management of symptoms [18]. After the concept of "symptom cluster" was put forward, research on disease management has gradually shifted from single symptoms to symptom clusters, but at present, most of the research focuses on symptom clusters of cancer patients [19–21]. Although some researchers have explored the symptom clusters of MHD patients, the formed symptom clusters are overlapping, ambiguous, and lacking in specificity [22]. Currently, most older ESRD patients choose HD as their treatment method [23]. Because the body functions and cognition of older patients are declining, it is common for multiple related symptoms to occur at the same time during the process of dialysis, which seriously affects the therapeutic effectiveness. According to a study [24], the one-year dialysis mortality rate of older patients over 65 years old is 54.5%, and the mortality rate of older HD patients gradually increases with age. However, after consulting the literature, it was found that the research on symptom clusters of older HD patients is very limited. Therefore, the management of symptom clusters of older MHD patients is an important field of research and innovation.

Although the introduction of the concept of symptom clusters is beneficial for the clustered management of symptoms, the absence of a distinction between primary and secondary relationships can lead to a lack of pertinence in management, resulting in poor efficiency [19]. In order to solve this problem, we need to conduct network analysis of patients' symptoms to find the patients' core symptoms, bridge symptoms, and sentinel symptoms, so that we can manage patients' symptom clusters in a targeted way. Symptom network is a quantitative study of the network structure, nodes, and network indicators composed of individual symptoms based on the complex network analysis system, and provides targets for precise intervention [21]. Symptom network has the function of dimension reduction; for example, it can visualize the relationship between symptoms through the network and show how symptoms within one symptom cluster relate to symptoms within another symptom cluster, thereby linking clusters of symptoms together [18, 25]. Moreover, it can guide healthcare workers and researchers in identifying core symptoms, bridging symptoms, and sentinel symptoms, focusing on micro interactions between symptoms and between clusters of symptoms [26]. So far, the study of symptom network has been carried out mostly in patients with cancer and mental disorders [26] but rarely in older MHD patients, who have a heavy burden of symptoms after dialysis. From the perspective of evaluating the mechanism of interaction between symptoms, it is not clear what the core, bridge, and sentinel symptoms of MHD patients are. This has to be clarified so as to develop a sound symptom management strategy.

Therefore, the purposes of this study were to (1) examine the occurrence of symptoms in older patients with MHD, and analyze and create symptom clusters; (2) generate symptom networks experienced by older patients with MHD and explore core, bridge, and sentinel symptoms; and (3) analyze the relationship between symptoms and symptom clusters of patients so as to provide evidence for the construction of scientific and efficient symptom management for patients.

## Methods

### Participants

This was a cross-sectional study, and the study report was drafted based on the STROBE statement. Older MHD patients in a tertiary hospital in Shanghai were selected as study participants from January 2022 to March 2022. Inclusion criteria were as follows [20]: (1) age: ≥ 60 years old; (2) routine HD ≥ 3 months; (3) stable dialysis cycle at 2–3 times/week; (4) symptom survey on symptoms that occurred during the dialysis period in patients; (5) voluntarily participation with informed consent. Exclusion criteria were as follows: (1) severe disease and inability to cooperate with the survey; (2) past mental disorder or language disorder. This study complied with the principle of volunteerism. All participants provided informed consent and voluntarily participated in this study.

### Measures

#### General condition questionnaire for older maintenance hemodialysis patients

Sociodemographic data included ethnicity, gender, age, occupational status, marital status, education level, per capita monthly income of family (yuan), medical payment method, residential status, dialysis duration (in months), general dialysis time, primary disease, and complications. Disease clinical information included urea clearance index, urea reduction ratio, serum creatinine, blood uric acid, blood beta-2 microglobulin, hemoglobin, albumin, proalbumin, transferrin, blood potassium, blood phosphorus, and blood calcium.

#### Modified dialysis symptom scale

The modified dialysis symptom index was developed by Yan Peng, a Chinese researcher, based on the Dialysis Symptom Index (DSI) developed by Weisbord et al., including the theory of unpleasant symptoms and adding frequency and severity dimensions to develop a scale that is suitable for dialysis patients in China. The symptom characteristics of the patients were evaluated based on four areas (presence/absence of symptoms; frequency [4-point Likert scale, a scale of 1–4, with higher scores indicating higher frequencies], severity [4-point Likert scale, a scale of 1–4, with higher scores indicating more severe symptoms], and distress [5-point Likert scale, a scale of 1–5, with higher scores indicating greater level of distress]) and included 30 symptom items. The Cronbach's α coefficients of the three dimensions of frequency, severity, and distress were 0.939, 0.948, and 0.958 respectively [27].

### Data collection procedures

Due to the impact of the COVID-19 outbreak on the progress of offline questionnaire collection, we adopted the WJX electronic version and paper version of questionnaire for surveys in this study. A preliminary survey was carried out on 10 older HD patients. After that, the questionnaire was revised. The revised electronic questionnaire contained 44 questions (including 14 general information questions and 30 dialysis symptom questions). The HD room nurse collected clinical data at corresponding time points based on the patient’s survey time.

We collected 29 online electronic questionnaires from January 24 to 26. One questionnaire was invalid, as it was a repeated questionnaire from a patient, and was removed. A total of 144 questionnaires were collected from January 28 to March 2. There were 139 valid questionnaires, and five questionnaires were invalid because the dialysis period was shorter than three months. The invalid questionnaires were removed.

Due to the impact of the COVID-19 outbreak in China, we were only able to collect questionnaires in designated hospitals, and the included patients had to meet the inclusion and exclusion criteria. Therefore, a total of 173 questionnaires were collected, of which 167 were valid, and invalid questionnaires were eliminated. The valid questionnaire recovery rate was 96.53%.

### Data analysis

#### Sample size estimation

In this study, we investigated a total of 30 symptoms in patients. The required sample size [28] was 5–10 times the number of variables, and considering a 10%–15% attrition rate, the calculated sample size was at least 165 cases. In order to manage the patients’ symptom clusters in a targeted manner, we conducted network analysis. The sample size of the network analysis was estimated to be 435 [29]. Referring to related studies [30], the results of the network analysis need to be evaluated considering other indexes such as stability besides the sample size. If other indexes perform well, the results could be accepted.

#### Analysis of types of symptom clusters

Excel 2019 was used for data entry. SPSS version 26.0 (IBM Corp., Armonk, NY, USA) was used for statistical analysis. Frequency, percentage, mean, and standard deviation were used to describe the demographic data and symptom occurrence of patients. In the process of data analysis, we excluded uncommon symptoms (prevalence < 10%) [31–33], including decreased interest in sex and difficulty becoming sexually aroused, from the analysis in order not to bias the results. Therefore, we actually included 28 symptoms in the analysis. After adjustment, it was calculated that we needed a sample size of 161 cases for factor analysis of symptom clusters. The total score of symptom experience was used to create symptom clusters. We used the spindle factorization method combined with the maximum variance rotation method to extract eigenvalues > 1. After 25 iterations, symptoms with factor load > 0.50 were grouped into symptom clusters.

#### Symptom network and node centrality

JASP version 0.15.0 (JASP Team) [34] was used for undirected network analysis. Since we actually included 28 symptoms in the analysis, the calculated sample size was 378 cases. Although our sample size of 167 did not reach the ideal sample size for network analysis, according to the relevant literature [30], the reliability of network analysis depends not only on whether the sample size meets the ideal sample size but also on the stability and accuracy of the results. According to the stability and accuracy of the results, our network analysis still has certain value. Symptoms were the nodes in the network, and edges represent the independent relationship between two nodes in the network. Edge thickness represents the magnitude of the relationship [35]. At the same time, the centrality markers of the model were calculated: strength, the sum of absolute value of correlation coefficient of edges; closeness, the inverse of the sum of distances between a node and all other nodes; betweenness, the number of times a node was on the shortest path between any other two nodes.

#### Determination of core symptoms, bridge symptoms, and sentinel symptoms

Higher closeness means that the symptom is located at the central position in the symptom network and has closer relationships with other symptoms [36]. Meanwhile, higher strength means greater weight and greater importance in the symptom network [37]. Strength can be used to determine the core symptoms of a patient. The higher the betweenness, the greater the role of the symptom in symptom interactions, and the symptom can be deemed an important bridge symptom in the symptom network [36, 38]. High symptom prevalence but low centrality means that the symptom could be a sentinel symptom for other symptoms [39]. Stability and accuracy are two indicators that reflect how stable and how accurate the estimated networks are. The stability was evaluated by calculating the correlation stability coefficient. Usually, a correlation stability coefficient should be at least 0.25, and a correlation stability coefficient > 0.50 is considered good [38]. The accuracy of the estimated network connections was evaluated by calculating the 95% confidence intervals (CIs) of the edge weight values [39].

## Results

### Characteristics of participants

A total of 167 older MHD patients were included in this study (111 men and 56 women). The ages of the participants were 60 years and above, and the mean dialysis duration was 56.25 ± 51.75 months. Table 1 shows specific details.

**Table 1:** General demographic data of hemodialysis patients (*n* = 167)

**Item**	***n***	Total score of symptom experience $\overline{\boldsymbol{x}}$** ± s**
**Sociodemographic data**		
Gender		
Male	111	68.77 ± 45.96
Female	56	86.30 ± 45.97
Age (years)		
60–64	44	82.82 ± 51.17
65–69	53	66.15 ± 43.86
70–74	30	63.83 ± 36.96
75–79	16	87.13 ± 50.34
≥ 80	24	83.67 ± 48.89
Ethnicity		
Han Chinese	166	74.43 ± 46.62
Ethnic minority	1	111.00 ± 0.00
Employment status		
Employed	3	90.67 ± 86.15
Unemployed	164	74.36 ± 45.96
Marital status		
Married	138	74.36 ± 45.96
Divorced	4	86.25 ± 60.12
Widowed	20	83.40 ± 34.68
Unmarried	5	34.40 ± 31.34
Education level		
Primary school and below	30	76.70 ± 42.93
Middle school	47	80.79 ± 50.05
High school/technical high school	51	64.10 ± 46.78
Junior college and above	39	79.49 ± 43.95
Average household monthly income (RMB)		
< 3000	19	79.16 ± 56.11
3000–3999	21	78.86 ± 55.40
4000–4999	38	62.47 ± 34.91
≥ 5000	89	77.90 ± 46.41
Healthcare payment mode		
Health insurance	165	73.97 ± 46.13
NCMS	1	83.00 ± 0.00
Self-pay	1	179.00 ± 0.00
Residential status		
Solitary	13	73.69 ± 39.24
Staying with family	149	73.69 ± 39.24
Other	5	56.60 ± 82.25
General dialysis time		
Morning	78	82.04 ± 45.61
Afternoon	72	82.04 ± 45.61
Night	17	75.65 ± 49.18
Primary disease		
Glomerulonephritis	56	70.14 ± 43.37
Hypertension	71	77.73 ± 50.02
Diabetes	62	77.97 ± 45.68
Others	37	72.81 ± 46.84
Complications		
Cardiovascular disease	56	97.38 ± 45.11
Infection	8	113.63 ± 65.88
Anemia	74	86.45 ± 45.08
Secondary hyperthyroidism	40	86.27 ± 47.12
Other	10	65.70 ± 30.53
**Clinical information**		
Creatinine (μmol/L)		
High	167	74.65 ± 46.57
Uric acid (μmol/L)		
Low	2	110.00 ± 32.53
Normal	104	76.96 ± 47.05
High	59	70.03 ± 46.41
Prealbumin (g/L)		
Low	25	94.04 ± 51.02
Normal	132	71.79 ± 45.76
High	8	66.13 ± 38.75
Hemoglobin (g/L)		
Low	156	73.88 ± 45.40
Normal	11	85.64 ± 62.47
Ferritin (ng/mL)		
Low	2	64.00 ± 29.70
Normal	87	71.07 ± 43.54
High	76	79.54 ± 50.49
Serum potassium (mmol/L)		
Low	15	78.93 ± 37.19
Normal	139	75.32 ± 48.06
High	10	75.32 ± 48.06
Phosphorus (mmol/L)		
Low	5	89.40 ± 22.77
Normal	64	72.98 ± 44.21
High	96	75.40 ± 49.39
Calcium (mmol/L)		
Low	25	70.60 ± 38.58
Normal	130	73.96 ± 48.71
High	10	97.60 ± 34.54
Urea clearance index (Kt/V)		
Met requirement	54	72.41 ± 47.95
Did not meet the requirement	110	75.83 ± 46.45
Urea reduction ratio (URR)		
Met the requirement	29	77.17 ± 43.31
Did not meet the requirement	135	74.17 ± 47.68

### Occurrence of post-dialysis symptoms in older maintenance hemodialysis patients

The results analysis of this study found that an average of 10 symptoms will occur in older MHD patients during dialysis. The top three symptoms by prevalence were dry skin, itching, and dry mouth, with prevalence of 74.85%, 71.86%, and 70.66%, respectively. This was followed by fatigue or weakness (61.08%), trouble staying asleep (60.48%), constipation (53.89%), and trouble falling asleep (51.50%). In post-dialysis symptoms, the symptom with the highest frequency was dry skin (2.47 ± 1.61), the symptom with the highest severity was trouble staying asleep (1.47 ± 1.41), and the symptom with the greatest distress was itching (2.83 ± 1.99). Table 2 shows specific details.

**Table 2:** Symptoms of older hemodialysis patients (*n* = 167)

**Symptom**	Prevalence	**Frequency** (4-point Likert scale) $\overline{x}$** ± s**	Severity (4-point Likert scale) $\overline{x}$** ± s**	Distress (5-point Likert scale) $\overline{x}$** ± s**
Constipation	53.89%	1.54 ± 1.56	0.93 ± 1.02	1.56 ± 1.66
Nausea	35.93%	0.71 ± 1.07	0.57 ± 0.86	1.25 ± 1.81
Vomiting	27.54%	0.54 ± 0.97	0.43 ± 0.79	0.98 ± 1.71
Diarrhea	8.38%	0.16 ± 0.57	0.14 ± 0.50	0.24 ± 0.84
Decreased appetite	34.13%	1.02 ± 1.52	0.62 ± 0.97	1.17 ± 1.72
Muscle cramp	48.50%	0.94 ± 1.07	0.71 ± 0.89	1.53 ± 1.74
Swelling in legs	14.37%	0.26 ± 0.70	0.22 ± 0.58	0.38 ± 0.97
Shortness of breath	14.37%	0.30 ± 0.80	0.27 ± 0.71	0.49 ± 1.22
Dizziness	41.32%	0.84 ± 1.10	0.58 ± 0.84	1.24 ± 1.63
Restless legs	13.17%	0.31 ± 0.88	0.22 ± 0.66	0.44 ± 1.21
Numbness or tingling in feet	31.14%	0.75 ± 1.27	0.53 ± 0.90	0.95 ± 1.54
Fatigue	61.08%	1.87 ± 1.67	1.10 ± 1.05	2.08 ± 1.83
Cough	28.14%	0.64 ± 1.14	0.42 ± 0.76	0.80 ± 1.38
Dry mouth	70.66%	2.18 ± 1.60	1.28 ± 1.05	2.36 ± 1.73
Bone or joint pain	31.74%	0.87 ± 1.37	0.56 ± 0.95	1.10 ± 1.76
Chest pain	16.17%	0.33 ± 0.82	0.28 ± 0.72	0.53 ± 1.30
Headache	17.37%	0.31 ± 0.73	0.25 ± 0.59	0.48 ± 1.15
Muscle soreness	11.98%	0.24 ± 0.73	0.22 ± 0.65	0.33 ± 0.98
Difficulty concentrating	9.58%	0.19 ± 0.63	0.18 ± 0.57	0.26 ± 0.81
Dry skin	74.85%	2.47 ± 1.61	1.38 ± 1.08	2.08 ± 1.59
Itching	71.86%	2.14 ± 1.58	1.37 ± 1.17	2.83 ± 1.99
Worrying	30.54%	0.63 ± 1.03	0.43 ± 0.73	0.86 ± 1.38
Feeling nervous	23.35%	0.49 ± 0.97	0.35 ± 0.71	0.64 ± 1.23
Trouble falling asleep	51.50%	1.62 ± 1.71	1.34 ± 1.47	2.19 ± 2.29
Trouble staying asleep	60.48%	1.90 ± 1.67	1.47 ± 1.41	2.46 ± 2.20
Feeling irritable	32.34%	0.70 ± 1.11	0.51 ± 0.86	0.95 ± 1.48
Feeling sad	29.34%	0.60 ± 1.01	0.50 ± 0.86	0.92 ± 1.51
Feeling anxious	33.53%	0.68 ± 1.03	0.51 ± 0.80	0.99 ± 1.50

Prevalence refers to the prevalence of a certain symptom in this investigated population; frequency refers to the number of times a symptom occurred in the past week in that survey population, and the frequency, severity, and distress in the tables are represented by means

### Cluster analysis of post-dialysis symptom clusters in older maintenance hemodialysis patients

In the process of data analysis, we excluded uncommon symptoms (prevalence < 10%) [30–32], including decreased interest in sex and difficulty becoming sexually aroused, from the analysis. These symptoms do not reflect the critical symptoms in the cluster and may interfere with the analysis and result interpretation. Therefore, we actually included 28 symptoms in the analysis. In this study, principal axis factoring was used to extract five independent symptom clusters, and these symptom clusters were named based on the characteristics of symptoms in these symptom clusters. These symptom clusters were the mood symptom cluster (consisting of worrying, feeling anxious, feeling nervous, feeling sad, and feeling irritable), gastrointestinal discomfort symptom cluster (consisting of nausea and vomiting), sleep disorder symptom cluster (consisting of trouble falling asleep and trouble staying asleep), skin discomfort symptom cluster (consisting of itching and dry skin), and cardiopulmonary discomfort symptom cluster (consisting of chest pain, shortness of breath, and cough). Other symptoms were not classified, as the burden was low, and were used as individual symptoms for intervention. Table 3 shows the details. The average frequency of symptoms in sleep disorder symptom cluster, skin discomfort symptom cluster, gastrointestinal discomfort symptom cluster, and mood symptom cluster was 1.76 ± 1.51, 2.30 ± 1.38, 0.62 ± 0.96, and 0.62 ± 0.83, respectively; the average severity was 1.40 ± 1.34 1.37 ± 1.02, 0.50 ± 0.78, and 0.46 ± 0.67, respectively; and the average degree of distress was 2.33 ± 2.06, 2.46 ± 1.55, 1.12 ± 1.67 and 0.87 ± 1.12, respectively.

**Table 3:** Factor loading of total symptom score after hemodialysis in older maintenance hemodialysis patients (*n* = 167)

**Symptom**	**Mood symptom cluster**	**Gastrointestinal discomfort symptom cluster**	**Cardiopulmonary discomfort symptom cluster**	**Sleep disorder symptom cluster**	**Skin discomfort symptom cluster**
Worrying	0.848				
Feeling nervous	0.824				
Feeling anxious	0.821				
Feeling sad	0.613				
Feeling irritable	0.525				
Nausea		0.955			
Vomiting		0.780			
Chest pain			0.673		
Cough			0.591		
Shortness of breath			0.502		
Trouble staying asleep				0.795	
Trouble falling asleep				0.789	
Itching					0.827
Dry skin					0.663
Eigenvalue	6.628	2.162	1.712	1.626	1.379
Variance contribution rate (%)	23.867	7.192	4.906	4.846	3.754
Cumulative variance contribution rate (%)	23.867	31.059	35.965	40.812	44.566

### Network analysis of post-dialysis symptoms in older maintenance hemodialysis patients

**Fig. 1:**
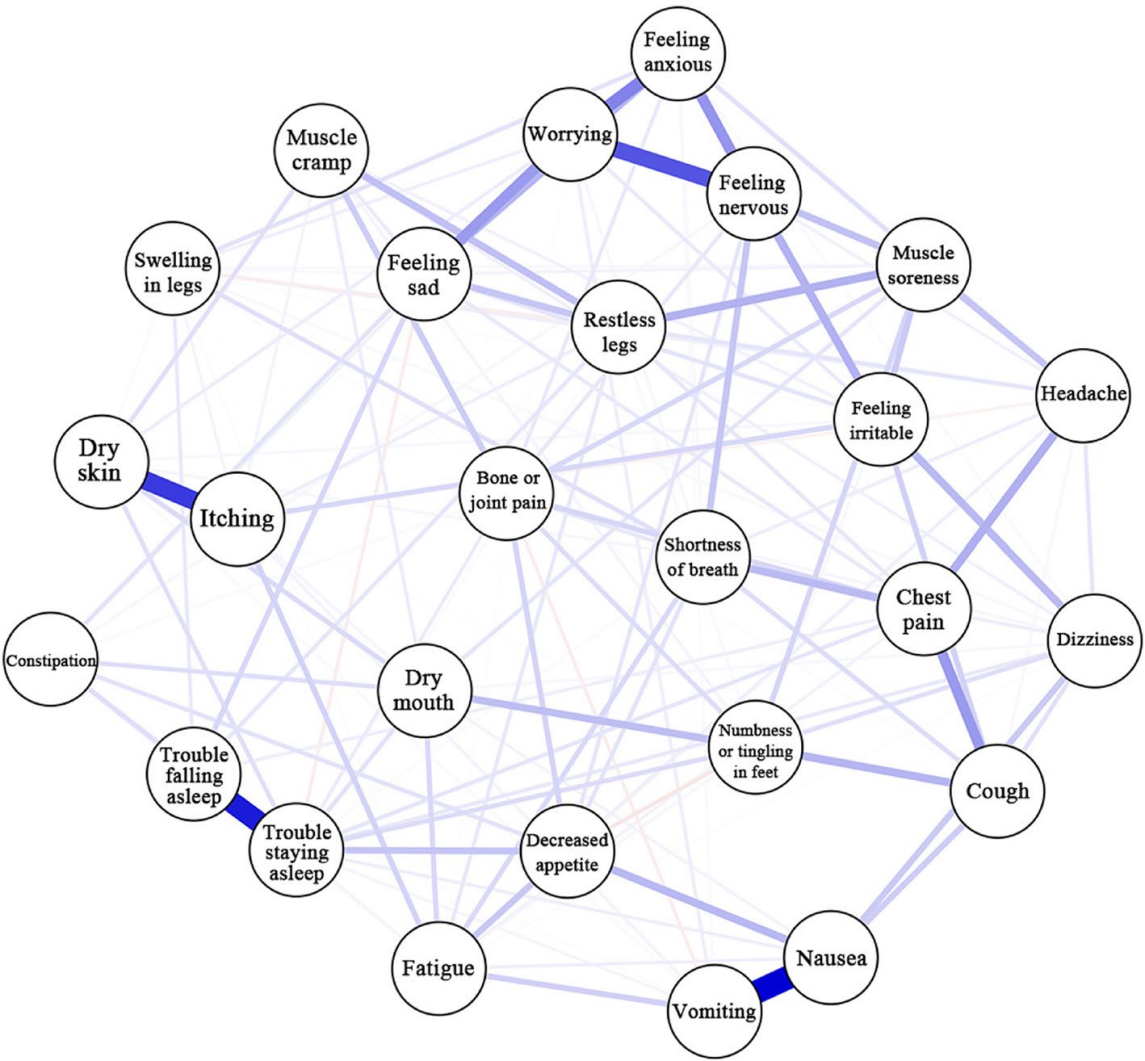
Network relationship map of post-dialysis symptoms in older maintenance hemodialysis patients

**Fig. 2:**
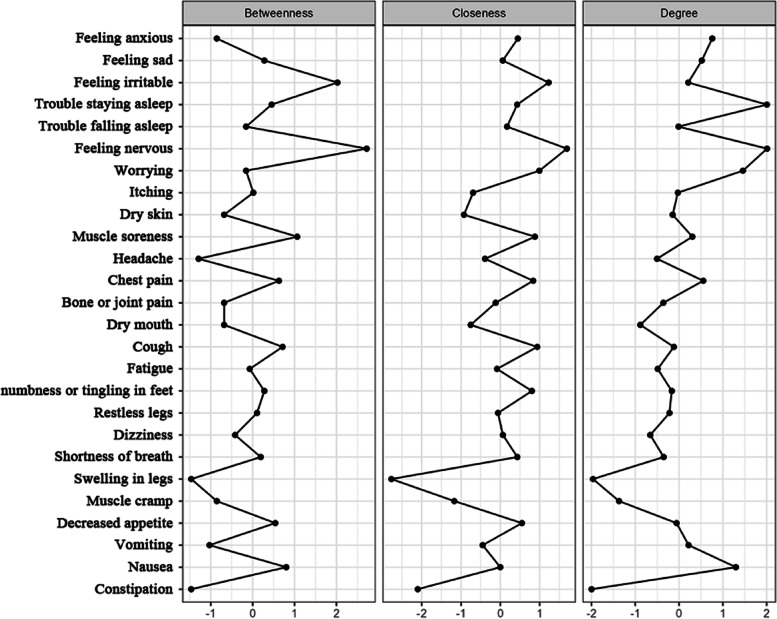
Centrality analysis of post-dialysis symptoms in older maintenance hemodialysis patients

We used network analysis to investigate the relationship between 28 common symptoms of HD patients. From the thickness of edges in symptom network, it could be seen that correlations were the strongest between feeling anxious, feeling nervous, feeling sad, and worrying; between nausea and vomiting; between trouble falling asleep and trouble staying asleep; and between dry skin and itching (Fig. 1), which was consistent with the symptom cluster analysis results. From the centrality markers, it was found that the symptoms with the highest strength were feeling nervous (*r*_s_ = 2.010) and trouble staying asleep (*r*_s_ = 2.005); symptoms with the highest betweenness were feeling nervous (*r*_b_ = 2.722) and feeling irritable (r_b_ = 2.024); symptoms with the highest closeness were feeling nervous (*r*_c_ = 1.692) and feeling irritable (*r*_c_ = 1.228) (Fig. 2). Feeling nervous had the highest closeness, were located in the center of the symptom network, and had the closest relationship with other symptoms [36]. Feeling nervous and trouble staying asleep had the highest strength, showing that their weights were high in the network, their influence was strong, and they were the most important symptoms in the symptom network [37]. Therefore, feeling nervous and trouble staying asleep are the core symptoms of older MHD patients. Feeling nervous and feeling irritable had high betweenness, showing that they had the greatest effects in symptom interactions and were important bridge symptoms in the symptom network [36]. Dry skin, itching, and dry mouth had the highest prevalence but low centrality, showing that they could be sentinel symptoms for other symptoms [39].

**Fig. 3:**
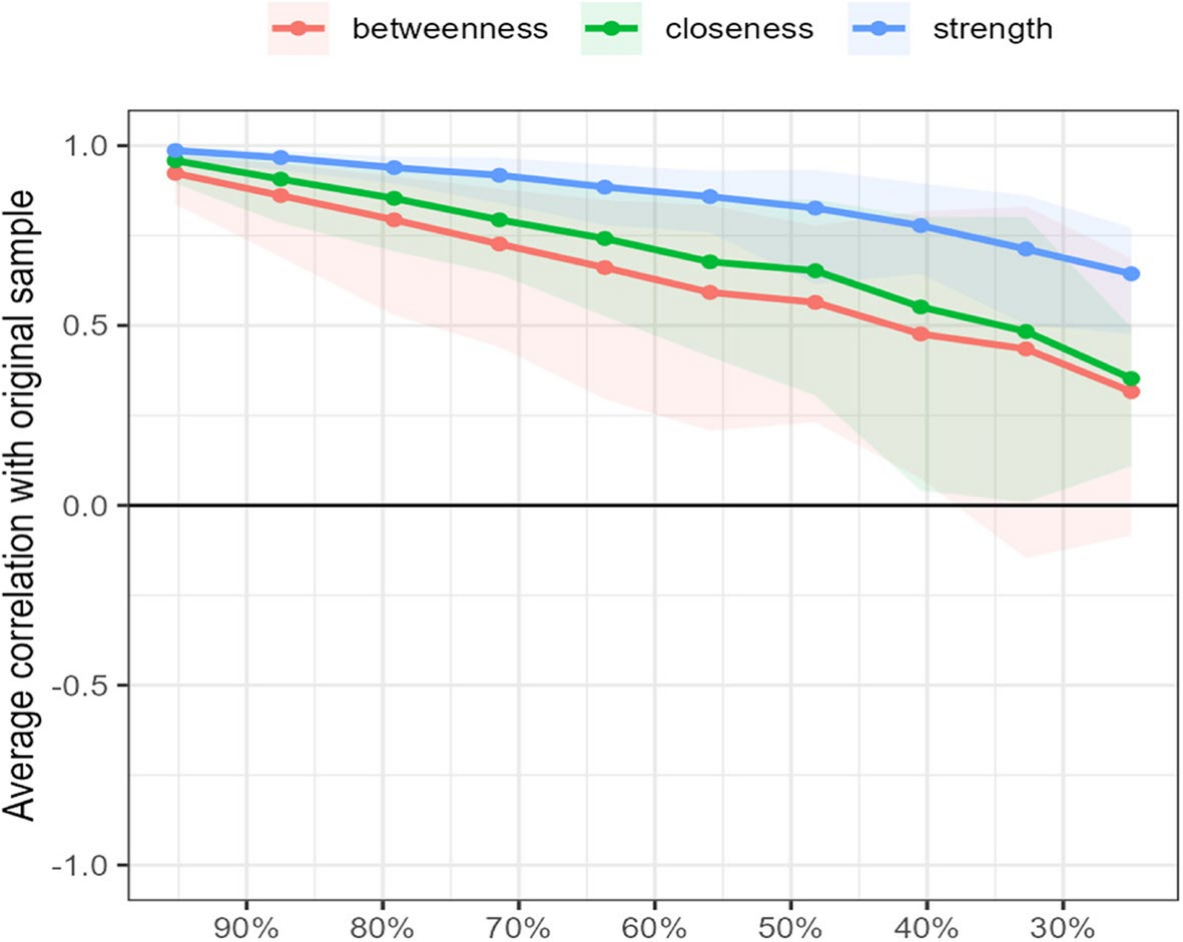
Correlation stability coefficient of symptom network in older maintenance hemodialysis patients

**Fig. 4:**
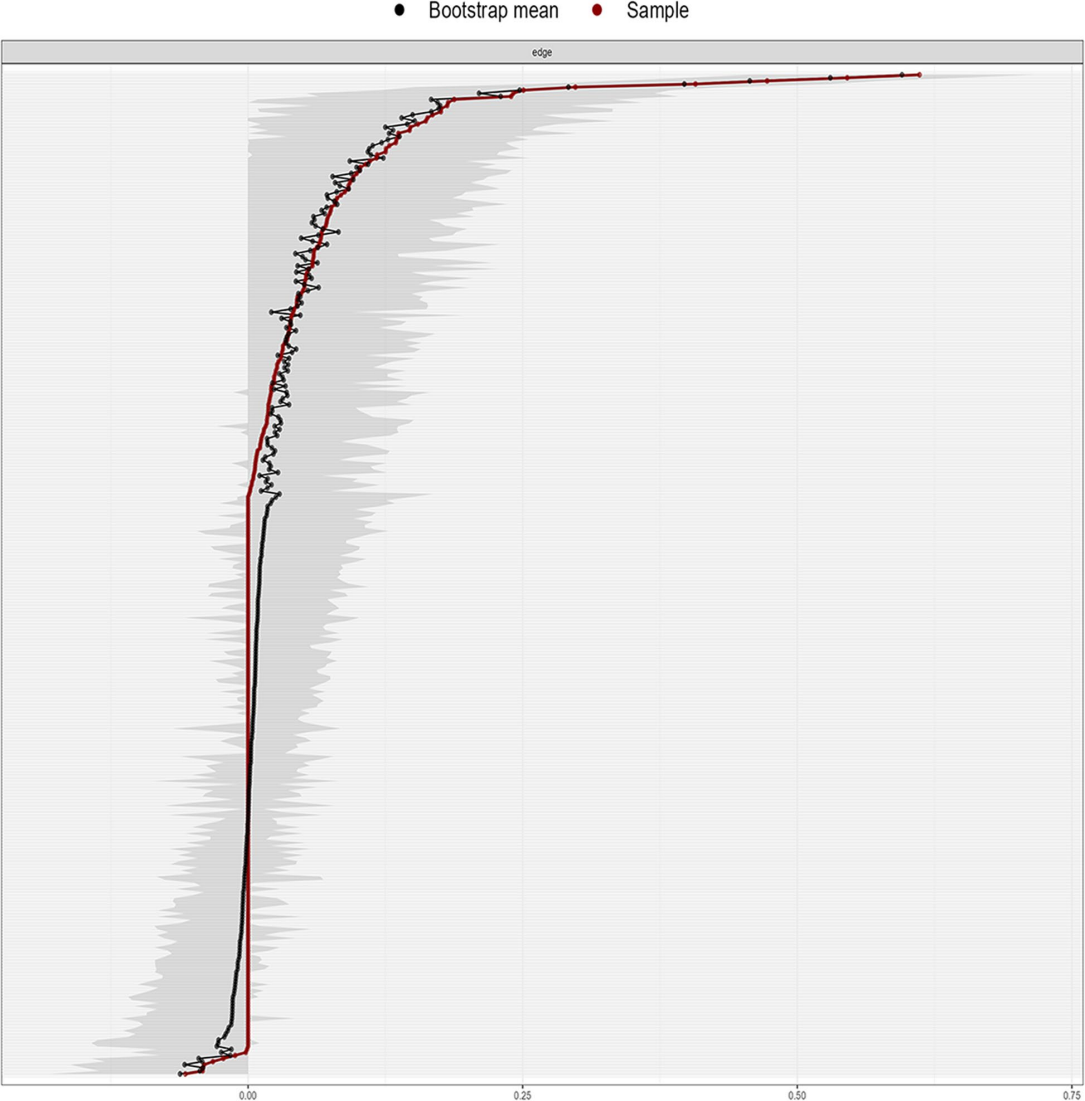
Bootstrap analysis results of the edge weights

Testing found that the correlation coefficients of strength in the symptom network were all greater than 0.5, suggesting that the network remained stable (Fig. 3). And calculation result also found that the bootstrapped CIs were small, which showed good accuracy of the network (Fig. 4).

## Discussion

### Severe post-dialysis symptom burden in older maintenance hemodialysis patients

The results of this study showed that patients on average developed 10 symptoms after dialysis. More than 70% of our participants had dry skin, itching, and dry mouth symptoms, which is similar to the results reported by Marques et al. (an average of 9 symptoms) [40]. Among the 30 symptoms investigated, the frequency and severity of dry skin, dry mouth, itching, trouble staying asleep, and fatigue were high (frequency: 2.47 ± 1.61, 2.18 ± 1.60, 2.14 ± 1.58, 1.90 ± 1.67, and 1.87 ± 1.67, respectively; severity: 1.38 ± 1.08, 1.28 ± 1.05, 1.37 ± 1.17, 1.47 ± 1.41, and 1.10 ± 1.05, respectively). Itching, trouble staying asleep, and dry skin caused the greatest distress to patients (2.83 ± 1.99, 2.46 ± 2.20, and 2.08 ± 1.59, respectively) and had a long duration. Some studies have shown that the decline in physical and social functions of HD patients after dialysis, coupled with strict restrictions on diet and fluid intake, leads to a significant decline in their quality of life [41]. To date, Chinese and international researchers have conducted many studies on symptom management in MHD patients [18], but most were focused on the management of individual symptoms, and there is a shortage of clinical nurse manpower. Therefore, the synergistic correlation of symptoms in a symptom cluster can be used clinically for more efficient and scientific management of symptoms [42]. However, corresponding targets must be found for symptom cluster management for targeted intervention. The selection of targets is vital to the results of symptom management. Therefore, this study conducted a network analysis of symptoms and symptom clusters and identified sentinel symptoms, core symptoms, and bridge symptoms in the network and elucidated the interaction mechanisms between symptoms, enabling targeted and feasible symptom (or cluster) management.

### Older maintenance hemodialysis patients have many symptom clusters during dialysis

In this study, five independent symptom clusters were extracted through exploratory factor analysis. These included mood symptom cluster, gastrointestinal discomfort symptom cluster, sleep disorder symptom cluster, skin discomfort symptom cluster, and cardiopulmonary discomfort symptom cluster. Among them, the symptom experience scores of patients were high for skin discomfort symptom cluster (6.13 ± 3.75), sleep disorder symptom cluster (5.49 ± 4.81), gastrointestinal discomfort symptom cluster (2.24 ± 3.31), and mood symptom cluster (1.95 ± 2.58), so more attention should be paid to the occurrence of these symptom clusters.

There is a certain correlation among the symptoms in the symptom clusters, and intervention of one symptom in the symptom clusters can also alleviate other symptoms [43]. There is evidence that treating one symptom can produce a "crossover" effect and reduce the discomfort of other symptoms in the cluster [44]. For example, the mood symptom cluster is composed of multiple negative mood symptoms, which can be systematically solved through psychological relief [45]. In the cluster of skin discomfort symptoms, dry skin is a common symptom of patients (74.85%). If not treated in time, it may cause skin itching in patients. External application of ointment can relieve dry skin and then relieve skin itching symptoms [46].

### Network analysis of post-dialysis symptoms in older maintenance hemodialysis patients

#### Dry skin, itching, and dry mouth are sentinel symptoms

The results of this study also showed that dry skin, itching, and dry mouth are sentinel symptoms in older MHD patients, indicating that other related symptoms are also present when these symptoms occur [47]. At the same time, the sentinel symptoms are also predictors of some other related symptoms [48]. Patients require strict water control during dialysis, as dialysis will metabolize excess water in the patient’s body, and ESRD patients are prone to serum calcium and phosphorus disturbances. Therefore, patients are prone to dry mouth, dry skin, and itching. The results of this study showed that the prevalence of dry mouth, dry skin, and itching was high (70.66%, 74.85%, and 71.86%, respectively) and caused great distress to patients (2.36 ± 1.73, 2.08 ± 1.59, and 2.83 ± 1.99, respectively). These results are consistent with the results of Kumar et al. [49, 50]. A previous study [17] showed that dry skin and itching correlated with decreased quality of life and depression. At the same time, dry skin and itching will cause or worsen other symptoms (particularly sleep disorder symptoms) and are independent predictors of mortality rate. Severe thirst will also cause poor sleep and negative emotions and increase water intake inappropriately in the patient. This causes excessive weight gain and body fluid overload during dialysis, resulting in discomfort symptoms. In current clinical practice, there is strict control of water intake, and patients with severe thirst are instructed to gargle with some warm water and receive acupuncture combined with traditional Chinese medicine treatment, which has demonstrated good results [51]. At present, most patients use skin moisturizers to alleviate dry skin and itching, with significant results [52]. Thirst, dry skin, and itching are apparent and are easier to control than other symptoms. Therefore, these symptoms have low centrality in the symptom network.

#### Trouble staying asleep and feeling nervous are core symptoms

The results showed that trouble staying asleep and feeling nervous are core symptoms in the symptom network model. Generally, it is believed that the effects of core symptoms are greater than those of edge symptoms in the network [53]. This suggests that early discovery and management of negative emotions is key to symptom management in MHD patients, as it has positive effects on the management of other symptoms (or clusters). The results of this study showed that the frequency and severity of trouble staying asleep are high, causing great distress to patients, which is similar to the results of Wang et al. [54]. From symptom network analysis, it can be seen that trouble staying asleep is intimately associated with sleep disorder, and both constitute the sleep disorder symptom cluster. Therefore, both symptoms have close synergism. Previous studies [54, 55] showed that sleep disorder is associated with the quality of life and long-term survival rate of patients. Poor long-term sleep quality will cause or worsen negative emotions and fatigue. At the same time, this can cause unstable blood pressure and blood glucose, increase cardiac burden, and even cause fatal complications. Therefore, trouble staying asleep greatly affects the occurrence of symptoms and treatment prognosis in older MHD patients and is a core symptom in these patients. Besides sleep disorder, patients will also experience many negative emotions during dialysis. A survey showed that patients are prone to nervousness, anxiety, and irritability during dialysis. These negative emotions will cause or worsen other symptoms, such as fatigue or sleep disorder [56, 57], and cause an immense disease burden for patients. At the same time, these emotions will decrease confidence in fighting the disease and the self-worth of patients [58], directly affecting dialysis quality and even increasing the mortality rate [59]. Therefore, feeling nervous has core effects on the occurrence of symptoms during dialysis in older MHD patients.

#### Feeling nervous and feeling irritable are bridge symptoms

The results of this study showed that feeling nervous and feeling irritable are bridge symptoms in the network, showing that they have a transmission role during their occurrence and are strong predictors of other symptoms [36]. At the same time, feeling nervous and feeling irritable are also core symptoms in older MHD patients, showing that these symptoms play extremely important roles in the entire symptom occurrence and progression process. A previous study [58, 60, 61] showed that long-term HD causes immense physical and mental suffering for patients. As dialysis causes patients to lose work and socializing opportunities and creates an immense financial burden for families, dialysis causes patients to face huge mental stress. At the same time, treatment results are not significant, the condition of older patients tends to worsen, and pain and itching cause irritation. This causes patients to be prone to nervousness, irritability, depression, and anxiety. The physical burden of MHD patients is intimately associated with their psychological burden. A high symptom burden tends to cause anxiety, sadness, nervousness, and depression. These negative emotions will accelerate heart rate, increase blood pressure, and cause or worsen other symptoms, such as fatigue and sleep disorder [56, 57]. This shows that negative emotions play a bridging role in the occurrence and progression of symptoms during dialysis in patients. Medical staff should focus on the role of feeling nervous and feeling irritable in the symptom network, encourage patients to actively report these symptoms, and carry out early intervention for related symptoms. Medical staff should pay close attention to mood changes in patients, promptly alleviate negative emotions, and help patients to solve difficulties in the treatment process. In addition, studies on the bridging roles of nervousness and irritability should be actively conducted, and their occurrence and the mechanisms through which they affect other symptoms should be examined to provide a basis for improving the precision and effectiveness of symptom management.

### The visual relationship between symptoms in the symptom network can be used to effectively manage symptom clusters

Among the symptom clusters, the symptom burden was high for skin discomfort cluster (6.13 ± 3.75), sleep disorder cluster (5.49 ± 4.81), gastrointestinal discomfort cluster (2.24 ± 3.31), and mood symptom cluster (1.95 ± 2.58). Network analysis found that symptoms in these four symptom clusters have close relationships and strong correlations, and attention should be paid to these symptom clusters.

Analysis found that dry skin and itching are sentinel symptoms of patients and constitute the skin discomfort symptom cluster. This means that the occurrence of this symptom cluster indicates that other related symptoms (or clusters) are present or about to occur, and intervention and early prevention of skin discomfort symptom cluster can alleviate the occurrence of other symptoms (or clusters). Worrying, feeling anxious, feeling nervous, feeling sad, and feeling irritable constitute the mood symptom cluster, and network analysis found that these mood symptoms have strong synergistic correlation. Among them, feeling nervous is core symptoms in patients, and feeling nervous and feeling irritable are bridge symptoms in the network. Therefore, intervention should be focused on feeling nervous and feeling irritable to alleviate the occurrence and progression of the mood symptom cluster. At the same time, the transmission effects of the mood symptom cluster should be utilized to effectively control the occurrence or worsening of other related symptoms (or clusters). Trouble staying asleep is also a core symptom of patients and constitutes the sleep disorder symptom cluster with difficulty falling asleep. Intervention should be focused on trouble staying asleep to effectively manage the sleep disorder symptom cluster and other related symptoms.

### Limitations

Due to the impact of the COVID-19 outbreak in China, the scope of the survey in this study was limited, and the sample size was insufficient, which may lead to slight bias in the results of the symptom network analysis. In addition, this was a cross-sectional survey, and the study scope was relatively limited, so we were not able to comprehensively clarify whether the symptom clusters of older MHD patients are stable over time. Therefore, researchers could strengthen the development of such studies in the future and carry out longitudinal studies on symptom clusters and symptom networks, mine the core and bridging effects of important symptoms, and identify the potential mechanisms of symptom clusters. This will provide a sufficient basis for precise and efficient symptom management.

## Conclusions

Older MHD patients have a high symptom burden after HD. Five independent symptom clusters were obtained from exploratory factor analysis, of which the clusters with the most severe symptom burdens were the gastrointestinal discomfort symptom cluster, sleep disorder symptom cluster, skin discomfort symptom cluster, and mood symptom cluster. Network analysis showed that symptoms in these four symptom clusters had strong correlations. Network analysis revealed that dry skin, itching, and dry mouth are sentinel symptoms in the network model. In clinical practice, communication with the patients should be strengthened, and attention should be paid to their chief complaints to identify thirst, dry skin, and itching early for intervention. Attention should also be paid to the evaluation of other related symptoms so as to achieve early intervention and prevention. Feeling nervous and trouble staying asleep are core symptoms of older MHD patients. Hence, clinical staff should pay attention to the occurrence of trouble staying asleep and feeling nervous in patients and provide prompt psychological and drug interventions to effectively control the occurrence or worsening of other related symptoms. Feeling nervous and feeling irritable are bridge symptoms in this symptom network model, and intervention in these symptoms should be prioritized in clinical practice to reduce their negative effects on other related symptoms. In clinical practice, we should pay attention to these important symptom clusters to effectively reduce the symptoms and treatment burdens of patients.

## Supplementary Information

**Additional file 1.**

## Data Availability

The datasets used and analyzed during the current study are available from the corresponding author on reasonable request.

## Abbreviations

K-NET: China Kidney Disease Network
DSI: Dialysis symptom index
ESRD: End-stage renal disease
MHD: Maintenance hemodialysis
URR: Urea reduction ratio

## References

[1] Himmelfarb J, Vanholder R, Mehrotra R, Tonelli M (2020). The current and future landscape of dialysis. Nat Rev Nephrol.

[2] Thurlow JS, Joshi M, Yan G, Norris KC, Agodoa LY, Yuan CM, Nee R (2021). Global epidemiology of end-stage kidney disease and disparities in kidney replacement therapy. Am J Nephrol.

[3] International Society of Nephrology (2019). Global kidney health atlas.

[4] Lee HJ, Son YJ (2021). Prevalence and associated factors of frailty and mortality in patients with end-stage renal disease undergoing hemodialysis: a systematic review and meta-analysis. Int J Environ Res Public Health.

[5] Karasneh R, Al-Azzam S, Altawalbeh SM, Alshogran OY, Hawamdeh S (2020). Predictors of symptom burden among hemodialysis patients: a cross-sectional study at 13 hospitals. Int Urol Nephrol.

[6] Flythe JE, Hilliard T, Lumby E, Castillo G, Orazi J, Abdel-Rahman EM, Pai AB, Rivara MB, St Peter WL, Weisbord SD, Wilkie CM, Mehrotra R, Kidney Health Initiative Prioritizing Symptoms of ESRD Patients for Developing Therapeutic Interventions Stakeholder Meeting Participants (2019). Fostering innovation in symptom management among hemodialysis patients: paths forward for insomnia, muscle cramps, and fatigue. Clin J Am Soc Nephrol.

[7] Wang X, Shi Q, Mo Y, Liu J, Yuan Y (2022). Palliative care needs and symptom burden in younger and older patients with end-stage renal disease undergoing maintenance hemodialysis: a cross-sectional study. Int J Nurs Sci.

[8] Antari GAA, Widyanthari DM (2020). Symptom burden and health-related quality of life in hemodialysis patients. Enferm Clin.

[9] Weng JM, Huang BH, Zhang WC, Chen WS, Shi J, Chen J, Wang MJ (2019). The association between residual renal function and symptom burden in maintenance hemodialysis patients. Chin J Blood Purif.

[10] You AS, Kalantar SS, Norris KC, Peralta RA, Narasaki Y, Fischman R, Fischman M, Semerjian A, Nakata T, Azadbadi Z, Nguyen DV, Kalantar-Zadeh K, Rhee CM (2022). Dialysis symptom index burden and symptom clusters in a prospective cohort of dialysis patients. J Nephrol.

[11] Ng MSN, Miaskowski C, Cooper B, Hui YH, Ho EHS, Mo SKL, Wong SSH, Wong CL, So WKW (2020). Distinct symptom experience among subgroups of patients With ESRD receiving maintenance dialysis. J Pain Symptom Manage.

[12] Chauhan K, Wen HH, Gupta N, Nadkarni G, Coca S, Chan L (2022). Higher symptom frequency and severity after the long interdialytic interval in patients on maintenance intermittent hemodialysis. Kidney Int Rep.

[13] Fleishman TT, Dreiher J, Shvartzman P (2020). Patient-reported outcomes in maintenance hemodialysis: a cross-sectional, multicenter study. Qual Life Res.

[14] Sharma R, Sharma SC, Chalise P, Regmee J, Sharma S (2022). Anxiety and depression among patients with chronic kidney disease undergoing haemodialysis in a tertiary care centre: a descriptive cross-sectional study. JNMA J Nepal Med Assoc.

[15] Ye W, Wang L, Wang Y, Wang C, Zeng J (2022). Depression and anxiety symptoms among patients receiving maintenance hemodialysis: a single center cross-sectional study. BMC Nephrol.

[16] Wang LS, Tong H, Liu QQ (2021). Research progress of fatigue in maintenance hemodialysis patients. Evid-Based Nurs..

[17] Lopes MB, Karaboyas A, Sukul N, Tsuruya K, Al Salmi I, Asgari E, Alyousef A, Schaufler T, Walpen S, Menzaghi F, Pisoni R (2022). Utility of a single itch-related question and the skindex-10 questionnaire for assessing pruritus and predicting health-related quality of life in patients receiving hemodialysis. Kidney Med.

[18] Harris CS, Dodd M, Kober KM, Dhruva AA, Hammer MJ, Conley YP, Miaskowski CA (2022). Advances in conceptual and methodological issues in symptom cluster research: a 20-year perspective. ANS Adv Nurs Sci.

[19] Sun Y, Chen Q, Li Y, Wang J, Bazzano AN, Cao F (2022). Prenatal symptom cluster of psychopathology and associations with mindfulness and rumination: a network analysis. J Nerv Ment Dis.

[20] Zhou YT, Cai XX, Lin YM, Wang N, Wang WW (2021). Frailty and its influencing factors in senile maintenance hemodialysis patients. Chin J Gerontol.

[21] Yang ZF, Zhu Z, Hu Y, Wen H, Zhang L, Fu YF, Wu B (2022). A review of network approach in symptom management. J Nurs Sci.

[22] Chen MC, Ho YF, Lin CC, Wu CC (2021). Development and testing of the hemodialysis symptom distress scale (HSD-22) to identify the symptom cluster by using exploratory factor analysis. BMC Nephrol.

[23] Okazaki M, Inaguma D, Imaizumi T, Hishida M, Kurasawa S, Kubo Y, Kato S, Yasuda Y, Katsuno T, Kaneda F, Maruyama S (2020). Impact of old age on the association between in-center extended-hours hemodialysis and mortality in patients on incident hemodialysis. PLoS ONE.

[24] Wachterman MW, O'Hare AM, Rahman OK, Lorenz KA, Marcantonio ER, Alicante GK, Kelley AS (2019). One-year mortality after dialysis initiation among older adults. JAMA Intern Med.

[25] Kalantari E, Kouchaki S, Miaskowski C, Kober K, Barnaghi P (2022). Network analysis to identify symptoms clusters and temporal interconnections in oncology patients. Sci Rep.

[26] Zhu Z, Xing W, Hu Y, Wu B, So WKW (2021). Paradigm shift: Moving from symptom clusters to symptom networks. Asia Pac J Oncol Nurs.

[27] Hao YH. Study of symptoms in hemodialysis patients. Master thesis. Beijing: Peking Union Medical College; 2016.

[28] Zhu LL, Jiang XL, Peng WX, Cheng JX, Zuo QT (2022). Longitudinal analysis of symptom clusters in chemotherapy patients after breast cancer surgery. J Nurs Sci.

[29] Epskamp S, Fried EI (2018). A tutorial on regularized partial correlation networks. Psychol Methods.

[30] Epskamp S, Borsboom D, Fried EI (2018). Estimating psychological networks and their accuracy: a tutorial paper. Behav Res Methods.

[31] Hao J, Gu L, Liu P, Zhang L, Xu H, Qiu Q, Zhang W (2021). Symptom clusters in patients with colorectal cancer after colostomy: a longitudinal study in Shanghai. J Int Med Res.

[32] Ye H, Zalesky A, Lv J, Loi SM, Cetin-Karayumak S, Rathi Y, Tian Y, Pantelis C, Di Biase MA (2021). Network analysis of symptom comorbidity in schizophrenia: relationship to illness course and brain white matter microstructure. Schizophr Bull.

[33] Rha SY, Lee J (2021). Stable symptom clusters and evolving symptom networks in relation to chemotherapy cycles. J Pain Symptom Manage.

[34] JASP team. JASP (Version 0.15) [Computer software]; 2021. https://jasp-stats.org/previous-versions/.

[35] Zhu Z, Wen H, Yang Z, Han S, Fu Y, Zhang L, Hu Y, Wu B (2021). Evolving symptom networks in relation to HIV-positive duration among people living with HIV: a network analysis. Int J Infect Dis.

[36] Ye YX, Qin L, Zeng K, Liang JW, Zhang LL (2022). Identifying core symptoms and symptom clusters in patients during intermittent period of cancer therapy. J Nurs Sci.

[37] Feng ZT, Zheng SS, Li X, Zhu H, Yin DQ, Ning YZ, Jia HX (2022). Relationship between Upper-heat and Lower-cold syndrome of generalized anxiety disorder based on hierarchical clustering and complex symptom network. J Cap Med Univ.

[38] Papachristou N, Barnaghi P, Cooper B, Kober KM, Maguire R, Paul SM, Hammer M, Wright F, Armes J, Furlong EP, McCann L, Conley YP, Patiraki E, Katsaragakis S, Levine JD, Miaskowski C (2019). Network analysis of the multidimensional symptom experience of oncology. Sci Rep.

[39] Zhu Z, Sun Y, Kuang Y, Yuan X, Gu H, Zhu J, Xing W (2023). Contemporaneous symptom networks of multidimensional symptom experiences in cancer survivors: a network analysis. Cancer Med.

[40] Ng MSN, Wong CL, Choi KC, Hui YH, Ho EHS, Miaskowski C, So WKW (2020). Mixed methods study of symptom experience in patients with end-stage renal disease. Nurs Res.

[41] Ng MSN, Wong CL, Ho EHS, Hui YH, Miaskowski C, So WKW (2020). Burden of living with multiple concurrent symptoms in patients with end-stage renal disease. J Clin Nurs.

[42] Ali S, Zheng J, Xiang JM, Gao LL (2021). Study on symptom clusters and influencing factors of pregnant women in the third trimester. J Nurs Sci.

[43] Zhang W. The symptom clusters and its influence factors among inpatients with gastrointestinal cancer. Master thesis. Anhui: Anhui Medical University; 2016.

[44] Miaskowski C, Barsevick A, Berger A, Casagrande R, Grady PA, Jacobsen P, Kutner J, Patrick D, Zimmerman L, Xiao C, Matocha M, Marden S (2017). Advancing symptom science through symptom cluster research: expert panel proceedings and recommendations. J Natl Cancer Inst.

[45] Chen Y, Ding J, Li C, Wu T, Li Q, Chen R, Zhou J (2022). Study on nursing effect of psychological intervention on uremic hemodialysis patients. Comput Math Methods Med.

[46] Ooi K (2021). Onset mechanism and pharmaceutical management of dry skin. Biol Pharm Bull.

[47] Xia C, Duan PB, Yang L, Yang LP, Han SX, Wang AQ (2021). Symptom clusters and sentinel symptoms of gastric cancer patients undergoing postopera-tive chemotherapy. J Nurs.

[48] Xia C. A study of symptom cluster and its sentinel symptoms in gastric cancer patients receiving chemotherapy after operation. Master thesis. Nanjing: Nanjing University of Traditional Chinese Medicine; 2021.

[49] Kumar MN, Swamy KNR, Thippeswamy HM, Giridhar K, Devananda D (2021). Prevalence of xerostomia in patients on haemodialysis: a systematic review and meta-analysis. Gerodontology..

[50] Liu Y, Sha YL, Li H, Zhang RZ (2020). Correlation study between classification of symptom clusters and laboratory indexes in hemodialysis patients. Chin Nurs Res.

[51] Cheng X, Hu JP, Luo F, Yu GJ (2021). Effect of acupuncture combined with linggui Zhugan Decoction on thirst in maintenance hemodialysis patients. WLD Chin Med.

[52] Yuan YY, Yang YJ, Zhang XX, Lu Y, Gan HY, Ying C (2022). Research progress on pruritus in hemodialysis patients. Chin Nurs Res.

[53] Costantini G, Epskamp S, Borsboom D, Perugini M, Mõttus R, Waldorp LJ, Cramer AOJ (2015). State of the aRt personality research: a tutorial on network analysis of personality data in R. J Res Pers.

[54] Wang YN, Ma ZF, Xiang J, Jiang YF (2019). Correlation and mediating effects of depression, sleep quality and fatigue in maintenance hemodialysis patients. Chin J Blood Purif.

[55] He S, Zhu J, Jiang W, Ma J, Li G, He Y (2019). Sleep disturbance, negative affect and health-related quality of life in patients with maintenance hemodialysis. Psychol Health Med.

[56] Lu HZ, Huang YL, Yang Z, Wang H, Chen GW, Zou BL (2021). Mediating role of anxiety between psychological flexibility and sleep quality in maintenance hemodialysis patients. Chin Nurs Res.

[57] Hou R, Song MF, Kang JJ, Guo YX (2021). Influencing factors of fatigue in in maintenance hemodialysis patients and their correlation with psychological resilience and hope. Chin Gen Pract.

[58] Chen ZW. Investigation of correlation between symptom burden,self-management behavior and quality of life in maintenance hemodialysis patients. Master thesis. Jilin: Yanbian University; 2018.

[59] Chen J, Liu L, Chen J, Ng MSN, Lou VWQ, Wu B, Jiang W, Jie Y, Zhu J, He Y (2021). The cross-lagged association between depressive symptoms and health-related quality of life in patients receiving maintenance hemodialysis: a three-wave longitudinal study. Qual Life Res.

[60] Uppal NN, Corona A, Fishbane S (2022). Pruritus in chronic kidney disease. Curr Opin Nephrol Hypertens.

[61] Liu J (2021). Correlations among disease management ability and self-efficacy and complicated cardiovascular disease of patients with maintenance hemodialysis. Med J Chin People's Health.

