# Supplementary material for: Exploration of symptom clusters during hemodialysis and symptom network analysis of older maintenance hemodialysis patients: a cross-sectional study
Source: BMC Nephrol. 2023 Apr 27;24:115. doi: 10.1186/s12882-023-03176-4 (PMC10132956; doi:10.1186/s12882-023-03176-4)
Supplement: Supplementary file 1 — Additional file 1. [file 12882_2023_3176_MOESM1_ESM.docx]

**Hemodialysis patient questionnaire**

**Demographic Sociological Data:**

1. Gender: [single choice] *

| ○Male |
| --- |
| ○Female |

2. Race: [fill in the blank]*

_________________________________

3. Age: [single choice]*

| ○From 60 to 64 |
| --- |
| ○From 65 to 69 |
| ○From 70 to 74 |
| ○From 75 to 79 |
| ○Greater than or equal to 80 |

4. Occupational status: [single choice] *

| ○On-the-job |
| --- |
| ○Non-employment |

5. Marital status: [single choice] *

| ○Married |
| --- |
| ○Divorce |
| ○Widowed |
| ○Unmarried |

6. Education level: [single choice]*

| ○Primary school or below |
| --- |
| ○Junior high school |
| ○High school or technical secondary school |
| ○College or above |

7. Per capita monthly income of family (yuan): [single choice] *

| ○Less than or equal to 3000 |
| --- |
| ○From 3000 to 3999 |
| ○From 4000 to 4999 |
| ○Greater than or equal to 5000 |

8. Medical payment method: [single choice]*

| ○Medical insurance |
| --- |
| ○New rural cooperative medical system |
| ○At your own expense |

9. Residential status [single choice] *

| ○Living alone |
| --- |
| ○Living with family |
| ○Others_________________ |

10. Dialysis duration (month(s)) [fill in the blank] *

_________________________________

11. General dialysis time: [single choice]

| ○Morning |
| --- |
| ○Afternoon |
| ○Night |

12. Primary disease: [single choice] *

| ○Glomerulonephritis |
| --- |
| ○Hypertensive nephropathy |
| ○Diabetes nephropathy |
| ○Others _________________ |

13. Complications: [multiple choice questions]

| □Cardiovascular disease |
| --- |
| □Infection |
| □Anemia |
| □Secondary hyperparathyroidism |
| □Others_________________ |

**Clinical Data**

1. Dry weight: [fill in the blank]

_________________________________

2. Weight change before and after dialysis (kg): [fill in the blank]

_________________________________

**Renal Function (3~7 questions)**

1. Urea clearance index [fill in the blank]

_________________________________

1. Urea reduction ratio [fill in the blank]

_________________________________

5. (before dialysis) Serum creatinine (Scr)（ μ Mol/L) [Fill in the blanks]

_________________________________

6. (before dialysis) Blood uric acid (UA) (mg/L)[fill in the blank]

_________________________________

7. (before dialysis) Blood beta-2 microglobulin (mg/L) [fill in the blank]

_________________________________

8. (before dialysis)Hemoglobin (Hb) (g/L) [fill in the blank]

_________________________________

**Nutritional Status Indicators (before dialysis) (8-10 questions)**

9. Albumin (Alb) (g/L) [fill in the blank]

_________________________________

10. Proalbumin (Pro-A) (mg/L) [fill in the blank]

_________________________________

11. Transferrin (TRF) (mg/L) [fill in the blank]

_________________________________

12. (before dialysis) Blood potassium (mmol/L) [fill in the blank]

_________________________________

13. (before dialysis) Blood phosphorus (mmol/L) [fill in the blank]

_________________________________

14. (before dialysis) Blood calcium (mmol/L) [fill in the blank]

_________________________________

**Modified Dialysis Symptom Assessment Scale**

Hello, the following table shows the physical and psychological symptoms that dialysis patients may have. Please read each symptom carefully and evaluate whether you have experienced these symptoms in the past week. If "Yes", continue to evaluate the frequency, severity and distress of the symptoms and tick "√" on the corresponding number.

**Assessment of dialysis symptoms in the past week ***

| In the past week: Have you had any of the following symptoms? | No | If there are:  How often does it come up? | | | | If there are:  How serious is it usually? | | | | If there are:  How much does it bother you or bother you? | | | | |
| --- | --- | --- | --- | --- | --- | --- | --- | --- | --- | --- | --- | --- | --- | --- |
|  |  | very few | sometimes | frequent | almost continuous | mild | moderate | heavy | extremely heavy | not at all | a little | somewhat | more | a lot |
| Constipation | ○ | ○ | ○ | ○ | ○ | ○ | ○ | ○ | ○ | ○ | ○ | ○ | ○ | ○ |
| Nausea | ○ | ○ | ○ | ○ | ○ | ○ | ○ | ○ | ○ | ○ | ○ | ○ | ○ | ○ |
| Vomiting | ○ | ○ | ○ | ○ | ○ | ○ | ○ | ○ | ○ | ○ | ○ | ○ | ○ | ○ |
| Diarrhea | ○ | ○ | ○ | ○ | ○ | ○ | ○ | ○ | ○ | ○ | ○ | ○ | ○ | ○ |
| Decreased appetite | ○ | ○ | ○ | ○ | ○ | ○ | ○ | ○ | ○ | ○ | ○ | ○ | ○ | ○ |
| Muscle cramp | ○ | ○ | ○ | ○ | ○ | ○ | ○ | ○ | ○ | ○ | ○ | ○ | ○ | ○ |
| Swelling in legs | ○ | ○ | ○ | ○ | ○ | ○ | ○ | ○ | ○ | ○ | ○ | ○ | ○ | ○ |
| Shortness of breath | ○ | ○ | ○ | ○ | ○ | ○ | ○ | ○ | ○ | ○ | ○ | ○ | ○ | ○ |
| Dizziness | ○ | ○ | ○ | ○ | ○ | ○ | ○ | ○ | ○ | ○ | ○ | ○ | ○ | ○ |
| Restless legs | ○ | ○ | ○ | ○ | ○ | ○ | ○ | ○ | ○ | ○ | ○ | ○ | ○ | ○ |
| Numbness or tingling in feet | ○ | ○ | ○ | ○ | ○ | ○ | ○ | ○ | ○ | ○ | ○ | ○ | ○ | ○ |
| Fatigue | ○ | ○ | ○ | ○ | ○ | ○ | ○ | ○ | ○ | ○ | ○ | ○ | ○ | ○ |
| Cough | ○ | ○ | ○ | ○ | ○ | ○ | ○ | ○ | ○ | ○ | ○ | ○ | ○ | ○ |
| Dry mouth | ○ | ○ | ○ | ○ | ○ | ○ | ○ | ○ | ○ | ○ | ○ | ○ | ○ | ○ |
| Bone or joint pain | ○ | ○ | ○ | ○ | ○ | ○ | ○ | ○ | ○ | ○ | ○ | ○ | ○ | ○ |
| Chest pain | ○ | ○ | ○ | ○ | ○ | ○ | ○ | ○ | ○ | ○ | ○ | ○ | ○ | ○ |
| Headache | ○ | ○ | ○ | ○ | ○ | ○ | ○ | ○ | ○ | ○ | ○ | ○ | ○ | ○ |
| Muscle soreness | ○ | ○ | ○ | ○ | ○ | ○ | ○ | ○ | ○ | ○ | ○ | ○ | ○ | ○ |
| Difficulty concentrating | ○ | ○ | ○ | ○ | ○ | ○ | ○ | ○ | ○ | ○ | ○ | ○ | ○ | ○ |
| Dry skin | ○ | ○ | ○ | ○ | ○ | ○ | ○ | ○ | ○ | ○ | ○ | ○ | ○ | ○ |
| Itching | ○ | ○ | ○ | ○ | ○ | ○ | ○ | ○ | ○ | ○ | ○ | ○ | ○ | ○ |
| Worrying | ○ | ○ | ○ | ○ | ○ | ○ | ○ | ○ | ○ | ○ | ○ | ○ | ○ | ○ |
| Feeling nervous | ○ | ○ | ○ | ○ | ○ | ○ | ○ | ○ | ○ | ○ | ○ | ○ | ○ | ○ |
| Trouble falling asleep | ○ | ○ | ○ | ○ | ○ | ○ | ○ | ○ | ○ | ○ | ○ | ○ | ○ | ○ |
| Trouble staying asleep | ○ | ○ | ○ | ○ | ○ | ○ | ○ | ○ | ○ | ○ | ○ | ○ | ○ | ○ |
| Feeling irritable | ○ | ○ | ○ | ○ | ○ | ○ | ○ | ○ | ○ | ○ | ○ | ○ | ○ | ○ |
| Feeling sad | ○ | ○ | ○ | ○ | ○ | ○ | ○ | ○ | ○ | ○ | ○ | ○ | ○ | ○ |
| Feeling anxious | ○ | ○ | ○ | ○ | ○ | ○ | ○ | ○ | ○ | ○ | ○ | ○ | ○ | ○ |
| Decreased interest in sex | ○ | ○ | ○ | ○ | ○ | ○ | ○ | ○ | ○ | ○ | ○ | ○ | ○ | ○ |
| Difficulty becoming sexually aroused | ○ | ○ | ○ | ○ | ○ | ○ | ○ | ○ | ○ | ○ | ○ | ○ | ○ | ○ |
